# Biology and Total Synthesis of n-3 Docosapentaenoic Acid-Derived Specialized Pro-Resolving Mediators

**DOI:** 10.3390/molecules29122833

**Published:** 2024-06-14

**Authors:** Amalie Føreid Reinertsen, Anders Vik, Trond Vidar Hansen

**Affiliations:** Department of Pharmacy, Section for Pharmaceutical Chemistry, University of Oslo, P.O. Box 1068, 0316 Oslo, Norway; a.f.reinertsen@farmasi.uio.no (A.F.R.); anders.vik@farmasi.uio.no (A.V.)

**Keywords:** specialized pro-resolving mediators, n-3 docosapentaenoic acid, n-3 DPA resolvins, n-3 DPA protectins, n-3 DPA maresins, T-series resolvins, stereoselective total synthesis, natural products

## Abstract

Research over the last 25 years related to structural elucidations and biological investigations of the specialized pro-resolving mediators has spurred great interest in targeting these endogenous products in total synthesis. These lipid mediators govern the resolution of inflammation as potent and stereoselective agonists toward individual G-protein-coupled receptors, resulting in potent anti-inflammatory activities demonstrated in many human disease models. Specialized pro-resolving mediators are oxygenated polyunsaturated products formed in stereoselective and distinct biosynthetic pathways initiated by various lipoxygenase and cyclooxygenase enzymes. In this review, the reported stereoselective total synthesis and biological activities of the specialized pro-resolving mediators biosynthesized from the polyunsaturated fatty acid n-3 docosapentaenoic acid are presented.

## 1. Introduction

### 1.1. Polyunsaturated Fatty Acids and Health Effects

**Figure 1:**
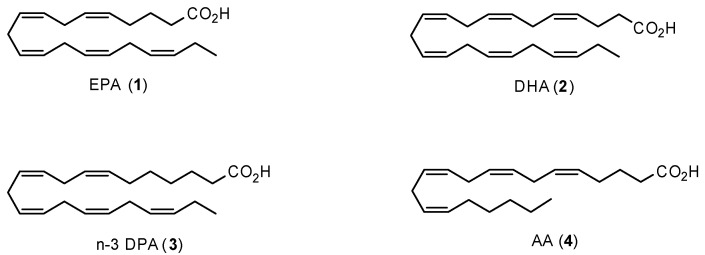
Chemical structures of the ω-3 PUFAs EPA (**1**), DHA (**2**), and n-3 DPA (**3**) as well as the ω-6 PUFA arachidonic acid (AA (**4**)).

A high dietary intake of ω-3 long-chain polyunsaturated fatty acids (PUFAs) is associated with many beneficial health effects [1]. These PUFAs include eicosapentaenoic acid (EPA, **1**), docosahexaenoic acid (DHA, **2**), and n-3 docosapentaenoic acid (n-3 DPA, **3**) (Figure 1). The ω-3 PUFAs are essential nutrients that cannot be biosynthesized by the human body in sufficient amounts and must therefore be obtained from the diet.

Moreover, the dietary ω-3 PUFAs have been shown to be associated with preventing a variety of inflammatory disorders [2], including cardiovascular diseases [3], rheumatoid arthritis [4], Alzheimer’s disease [5], asthma [6], and type 2 diabetes [7]. Until recently, no molecular basis or cellular mechanisms have been established for the health effects accounted for the ω-3 PUFAs. However, the diligent and continuous efforts led by Professor Charles N. Serhan and collaborators over the last 25 years have demonstrated that the three ω-3 PUFAs **1**–**3**, but also the ω-6 PUFA arachidonic acid (AA, **4**, Figure 1), are precursors for the enzymatically formed oxygenated products named specialized pro-resolving mediators (SPMs). SPMs potently down-regulate the inflammatory process and possess nanomolar pro-resolving bioactions [8,9,10]. Since uncontrolled inflammation is a common theme for the human diseases listed above, the pro-resolving and anti-inflammatory bioactions reported for the SPMs have attracted great interest in biomedical research [11]. SPMs are also highly interesting targets for stereoselective total synthesis, enabling drug discovery projects [12].

### 1.2. Inflammation, Resolution of Inflammation, and Lipid Mediators in Inflammation

The inflammatory process is an essential part of the protective response to tissue injury and infection by invading microbial pathogens [13]. The inflammatory response may be divided into acute and chronic inflammation, which are defined according to the nature of the inflammatory cells appearing in tissue [13]. Acute inflammation is further divided into the initiation phase and the resolution phase of inflammation. The former has the classic cardinal signs such as rubor (redness), calor (heat), tumor (swelling), and dolor (pain), described by Celsus in the 1st century [9], in addition to the loss of function, which was added by Rudolf Virchow in the 19th century [14]. Although the primary goal of the inflammation phase is to regain homeostasis [15], if kept uncontrolled, it may result in the development of a chronic state of inflammation. 

**Figure 2:**
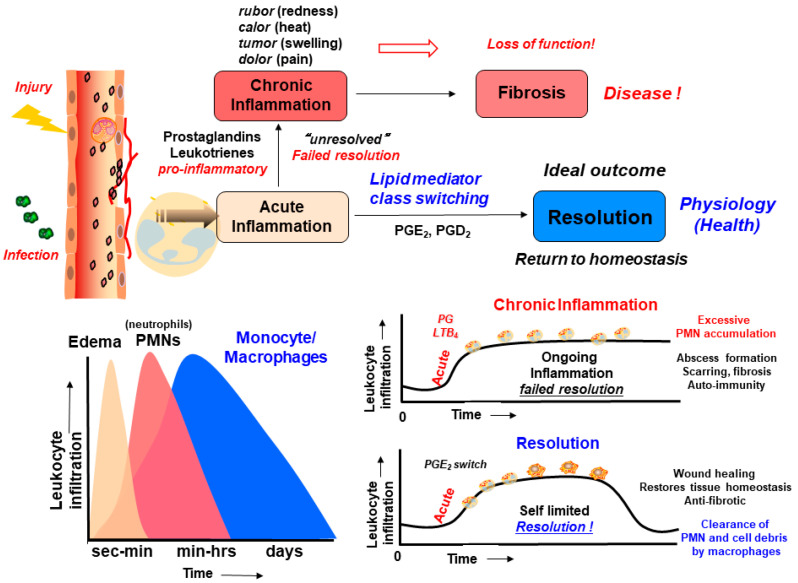
The figure illustrates the two major outcomes of the inflammatory process.

The course from initiation to the resolution of acute inflammation is illustrated in Figure 2 and shows the most central cell types in the different stages of inflammation. In the early stages of the inflammatory process, activated endothelial cells start to produce pro-inflammatory mediators, such as cytokines and chemokines, as well as chemoattractants like histamine and bradykinin. Additionally, pro-inflammatory lipid mediators, such as the leukotrienes (LTs) and prostaglandins (PGs), are biosynthesized from AA (**4**) after its release from the phospholipid membrane. These mediators increase vessel permeability, vasodilation, and the recruitment of leukocytes to the site of injury, leading to the classic cardinal signs of inflammation [9,13].

**Figure 3:**
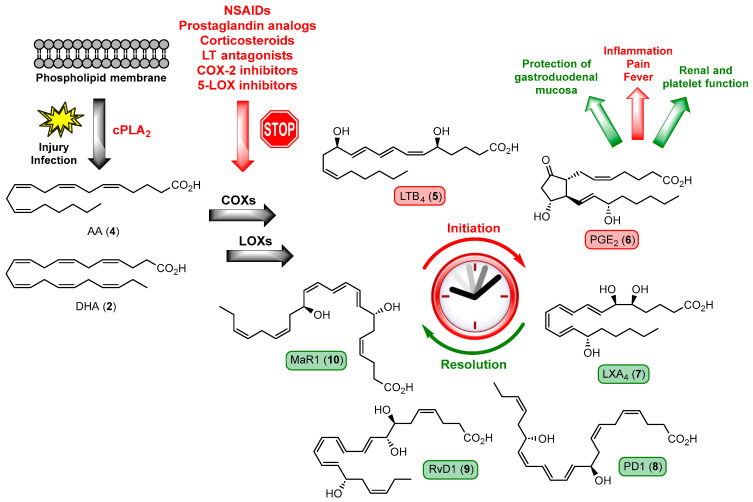
PUFAs, such as DHA (**2**) and AA (**4**), are released from the phospholipid membrane because of injury or infection, thus resulting in the biosynthesis of a variety of lipid mediators through the COX- and LOX pathways. The initiation of inflammation stimulates the biosynthesis of pro-inflammatory lipid mediators (LTB_4_ (**5**), PGE_2_ (**6**) followed by a lipid mediator class switch toward the biosynthesis of the anti-inflammatory and pro-resolving SPMs, e.g., LXA_4_ (**7**), PD1 (**8**), RvD1 (**9**), and MaR1 (**10**). The figure also highlights some anti-inflammatory drug classes that target the biosynthesis of lipid mediators, hence downregulating both the pro-inflammatory and the host protective roles of the prostaglandins. These traditional pharmaceuticals interrupt the normal resolution phase.

Basophils, eosinophils, neutrophils, monocytes, and lymphocytes are examples of leukocytes. The neutrophils, also known as the polymorphonuclear neutrophils (PMNs), are the most predominant of these cell types in the early stages of inflammation [16]. The PMNs are among the first cells to appear at the site of injury or infection and act as the host’s first line of defense, as these cells swarm to the inflamed tissue. The PMNs are essential for further acceleration of the inflammatory process by the production of inflammatory mediators, including cytokines, chemokines, lipid mediators, and growth factors [13]. The inflammatory response needs to be terminated once the incoming stimulus, caused by tissue injury or invasion of pathogens, has been defeated. During the termination of ongoing inflammation, also referred to as the resolution phase, PMNs are gradually being replaced by mononuclear cells, mainly monocytes (Figure 2). These cells differentiate into macrophages in the tissue and are often referred to as phagocytic cells, meaning they ingest foreign material and cell debris in a process named phagocytosis. Macrophages also clear apoptotic cells and cellular debris in a process named efferocytosis. Phagocytosis and efferocytosis are typical processes associated with SPMs during the resolution of inflammation. Another important event in the resolution phase of inflammation is the timely lipid mediator class switch, illustrated in Figure 3. Herein, the biosynthesis of the pro-inflammatory LTs and PGs, such as leukotriene B_4_ (LTB_4_, **5**) and prostaglandin E_2_ (PGE_2_, **6**), are diminished, and replaced by an enhanced biosynthesis of SPMs, such as lipoxin A_4_ (LXA_4_, **7**), protectin D1 (PD1, **8**), resolvin D1 (RvD1, **9**) and maresin 1 (MaR1, **10**), thus initiating resolution of inflammation [17]. The release of AA (**4**) from the cell membrane by cytosolic phospholipase A_2_ (cPLA_2_) is the first and overall rate-determining step in the biosynthesis of eicosanoids by effector and immune cells, as illustrated in Figure 3. This unbound form of intracellular AA (**4**) is rapidly converted in a cell type-specific manner by the cyclooxygenase (COX) or lipoxygenase (LOX) enzymes to generate lipid mediators, such as LTB_4_ (**5**) and PGE_2_ (**6**) [13], with pro-inflammatory properties.

Failure to reduce further neutrophil recruitment and clearance of apoptotic cells by macrophages may result in the development of a chronic state of inflammation [9,18]. Chronic inflammation is defined according to the accumulation of lymphocytes, macrophages, and plasma cells in the tissue and not by the duration of the inflammatory process [19]. When present in extravascular sites, these cells may lead to the secretion of a variety of factors, one example being tumor necrosis factor-β (TNF-β). Such factors activate fibroblasts and result in the production of cross-linked collagen, which may, in turn, lead to extensive collagenous scars [13]. This highly undesirable outcome of the acute inflammatory response has proved to be a part of the pathogenesis of various disorders [2,3,4,5,6,7,20]. As stated above, the ideal outcome of an inflammation is resolution, a process that is governed by SPMs. Thus, the active process of resolution of inflammation by SPMs biosynthesized from the ω-3 PUFAs EPA (**1**), DHA (**2**), and n-3 docosapentaenoic acid (n-3 DPA, **3**) is a dynamic and detailed programmed response, and not just a means of passive dilution of chemoattractants, as previously thought [13]. These active processes leading to the resolution of inflammation are considered a biomedical paradigm shift [21,22]. Resolving inflammatory exudate converts the ω-3 PUFAs **1**–**3** to families of structurally distinct signaling molecules, named resolvins, protectins, and maresins, while the ω-6 PUFA AA (**4**) forms lipoxins [8]. SPMs are agonists in the resolution phase of inflammation and exert their bioactions by stereoselective interaction with G-protein-coupled receptors (GPCRs), hence limiting the infiltration of PMNs and enhancing the clearance of apoptotic cells by phagocytosis [18]. The importance of SPMs in the resolution phase of inflammation may lead to the development of small organic molecular drugs that are not immunosuppressive [18,21] or constitute a new way to treat inflammation in the future based on the principles of resolution pharmacology [22,23,24]. 

### 1.3. Overview of Specialized Pro-Resolving Mediators

**Figure 4:**
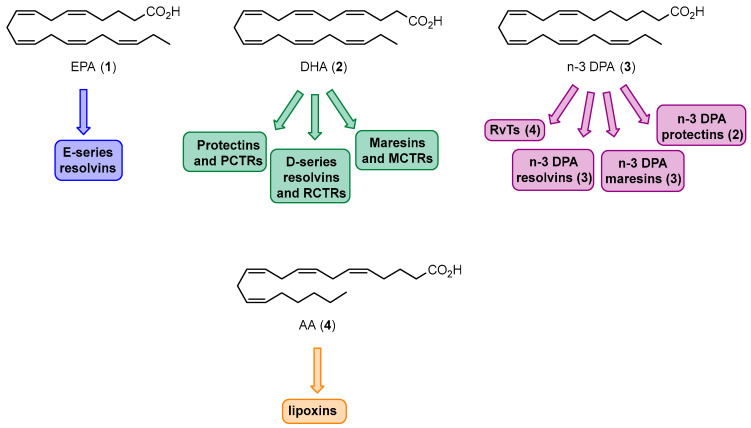
Overview of the different SPM families derived from the ω-3 PUFAs EPA (**1**), DHA (**2**), and n-3 DPA (**3**) and the ω-6 PUFA AA (**4**). The number of each reported n-3 DPA-derived SPM is provided in parentheses.

Research over the last three decades has led to the structural elucidations and biological investigations of the SPM families outlined in Figure 4. SPMs have shown potent and interesting biological effects in many human disease models and cell systems [25,26,27,28]. 

The resolvins biosynthesized from DHA (**2**) are termed D-series resolvins (RvDs), while EPA (**1**) gives rise to the E-series resolvins (RvEs). DHA (**2**) also gives rise to the protectins and maresins, including the sulfido-conjugated compounds called protectin conjugates in tissue regeneration (PCTRs), resolvin conjugates in tissue regeneration (RCTRs), and maresin conjugates in tissue regeneration (MCTRs) (Figure 4). The term “resolvins” reflects their resolving features during inflammation [29], and the “protectins” got their name due to their potent protective activity in inflammatory and neuronal systems [30]. The “maresins” were assigned this name due to their role as macrophage mediators that resolve inflammation [31]. Hamberg, Serhan, and Samuelsson reported in 1984 that AA (**4**) was biosynthesized into lipoxins A_4_ and B_4_ [32,33]. The n-3 DPA (**3**)-derived SPMs include the n-3 DPA resolvins, n-3 DPA maresins, n-3 DPA protectins [34], and the most recently identified 13-series resolvins (RvTs) [35]. 

### 1.4. Specialized Pro-Resolving Mediators Derived from n-3 DPA

In 2013, Dalli, Collas, and Serhan reported that n-3 DPA (**3**), like EPA (**1**) and DHA (**2**), is a substrate for 5-, 12-, and 15-LOX enzymes in mice and human leukocytes, leading to the discovery of the novel n-3 DPA-derived SPMs [34,35]. These SPMs share structural resemblance with the DHA-derived protectins, maresins, and resolvins, except for the absence of the C_4_–C_5_ *Z*-olefin in the n-3 DPA-derived respective protectins, maresins, and resolvins. Hence, these 12 SPMs are congeners of the DHA-derived SPMs. In addition, Dalli, Chiang, and Serhan reported investigations of the transcellularly biosynthesis using n-3 DPA (**3**) and COX-2 during neutrophil–endothelial cell interactions. These studies resulted in the identification of the four-membered 13-resolvins (RvTs) [35]. The naming originates from the first and common oxygenation step occurring at carbon thirteen [35]. The hitherto reported stereoselective total syntheses of the n-3 DPA SPMs are discussed below, but first, the individual families of the n-3 DPA-derived SPMs are presented. 

#### 1.4.1. Biosynthesis of n-3 DPA Protectins

**Scheme 1:**
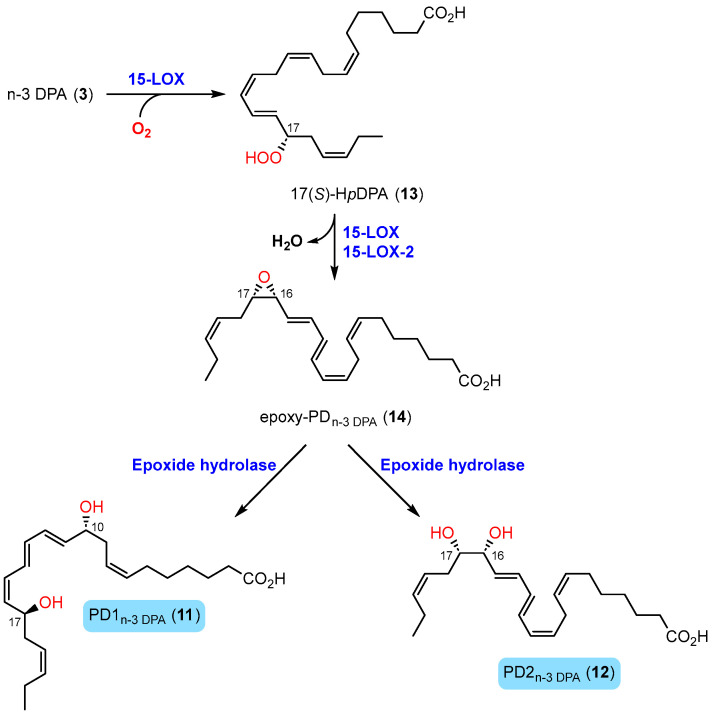
Biosynthesis of the n-3 DPA protectins **11** and **12**.

As their names suggest, PD1_n-3 DPA_ (**11**) and PD2_n-3 DPA_ (**12**) are both biosynthesized from n-3 DPA (**3**) [34,36,37]. 15-LOX activity on n-3 DPA (**3**) yields the known 17(*S*)-H*p*DPA (**13**) intermediate that is further subjected to 15-LOX and 15-LOX-2 to yield the epoxide intermediate **14** [36,37]. Different epoxide hydrolase activities lead to the formation of either PD1_n-3 DPA_ (**11**) or PD2_n-3 DPA_ (**12**) [37]. The biosynthetic pathways of n-3 DPA protectins, in contrast to the other n-3 DPA families of SPMs, have been studied in detail [36,37], as illustrated in Scheme 1.

#### 1.4.2. Biosynthesis of n-3 DPA Maresins

**Scheme 2:**
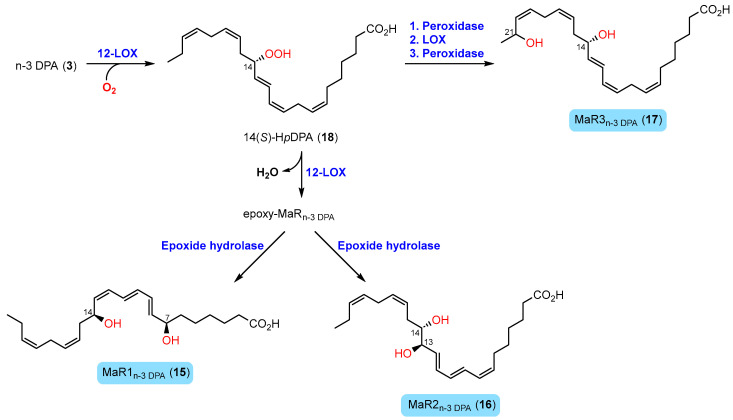
Proposed mechanism for the biosynthesis of the n-3 DPA maresins. The absolute configuration is provided where known.

The maresins derived from n-3 DPA (**3**) comprise MaR1_n-3 DPA_ (**15**), MaR2_n-3 DPA_ (**16**), and MaR3_n-3 DPA_ (**17**). n-3 DPA (**3**) is a substrate for 12-LOX, and this oxygenase forms the common and known intermediate 14(*S*)-H*p*DPA (**18**), as shown in Scheme 2. Enzymatic epoxidation of **18** by 12-LOX most likely forms an epoxide that undergoes ring opening by different epoxide hydrolases, producing either MaR1_n-3 DPA_ (**15**) or MaR2_n-3 DPA_ (**16**). Alternatively, the insertion of molecular oxygen at C_21_ in **18** and additional peroxidase activity forms MaR3_n-3 DPA_ (**17**). Of note, these biosynthetic pathways are presented based on prior knowledge [8,34] and have not been established by experiments. 

#### 1.4.3. Biosynthesis of n-3 DPA Resolvins

**Scheme 3:**
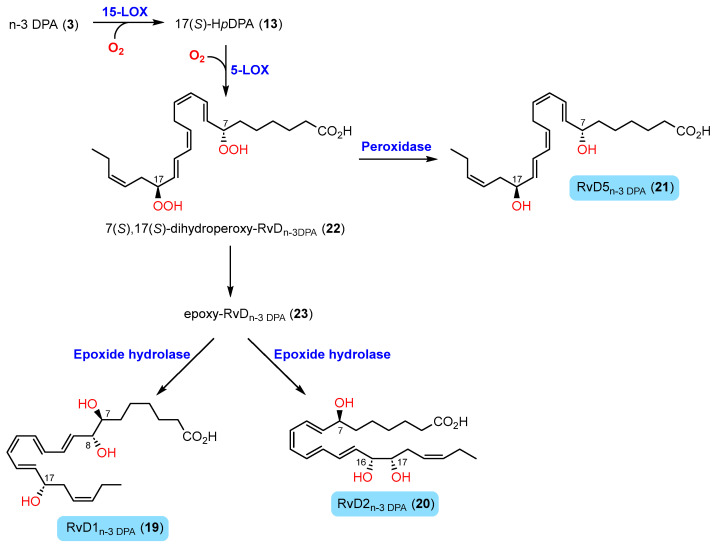
Proposed biosynthesis of the n-3 DPA resolvins **19**–**21**.

The n-3 DPA-derived resolvins consist of RvD1_n-3 DPA_ (**19**), RvD2_n-3 DPA_ (**20**), and RvD5_n-3 DPA_ (**21**). The proposed biosynthesis suggests the insertion of molecular oxygen to n-3 DPA (**3**) by 15-LOX to yield the common and known intermediate 17(*S*)-H*p*DPA (**13**), as shown in Scheme 3. Then, 5-LOX catalyzes the formation of another hydroperoxide in the C_7_ position to yield the intermediate 7(*S*),17(*S*)-dihydroperoxy-RvD_n-3 DPA_ (**22**). An enzymatic conversion yields an epoxy intermediate that is further hydrolyzed by different epoxide hydrolase enzymes to afford either RvD1_n-3 DPA_ (**19**) or RvD2_n-3 DPA_ (**20**). Alternatively, direct peroxidase activity on **22** gives rise to RvD5_n-3 DPA_ (**21**) [34]. As of today, no direct evidence is available for the biosynthesis presented in Scheme 3, but it should share great similarities to the biosynthesis of the DHA-congener resolvin D1 (**9**) [29,38] (see Figure 3).

A self-limited model of inflammation was applied to investigate tissue levels of n-3 DPA products during onset and resolution of inflammation [34]. In these studies, the concentration of RvD1_n-3 DPA_ (**19**) showed a bi-phasic profile by reaching a maximum during peak neutrophil infiltration and late into resolution. The peak level of RvD2_n-3 DPA_ (**20**) accorded with the onset of resolution, i.e., the point where PMN levels reach ~50% of transport maximum (T_max_). The level of RvD5_n-3 DPA_ (**21**) gradually increased over the course of inflammation resolution, with a maximum in the late stages of the resolution phase. 

#### 1.4.4. Biosynthesis of 13-Series Resolvins

**Scheme 4:**
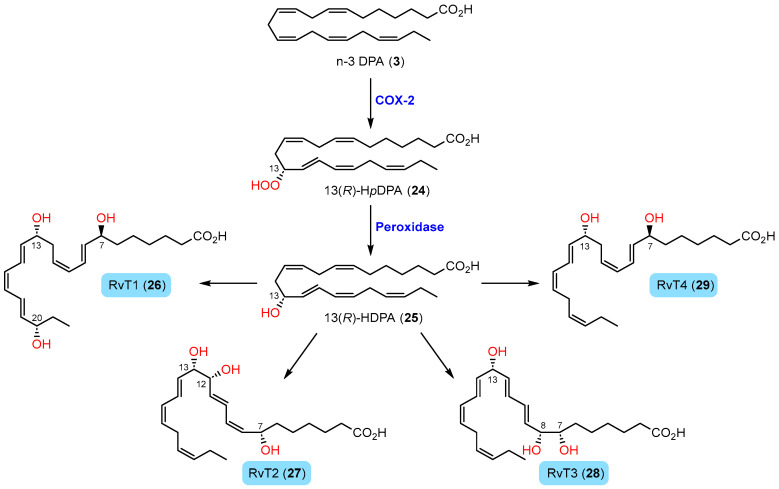
Proposed mechanism for the biosynthesis of the 13-series resolvins **26**–**29**.

**Scheme 5:**
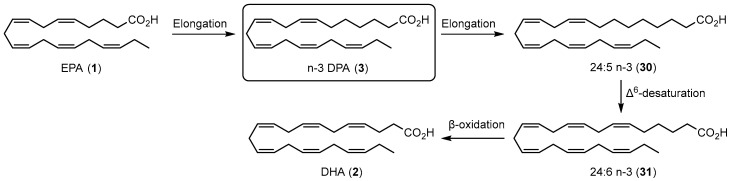
Outline of the mammalian biosynthesis of DHA (**2**) from EPA (**1**) via the intermediate n-3 DPA (**3**).

In 2015, a new series of n-3 DPA-derived resolvins was reported and termed 13-series resolvins [35]. Their names are supported by the common 13(*R*)-alcohol moiety in all four structures [39]. The biosynthesis of the RvTs commences with the insertion of molecular oxygen at C_13_ by COX-2 enzymes to yield 13(*R*)-H*p*DPA (**24**), as shown in Scheme 4. The peroxide intermediate **24** is then subjected to peroxidase enzymes to yield the 13(*R*)-alcohol **25** [40]. Different enzymatic activities on **25** form the four RvTs **26**–**29**. Interestingly, the biosynthetic formation of these SPMs was increased by atorvastatin via S-nitrosylation of the COX-2 enzymes and reduced by COX-2 inhibitors [35].

n-3 DPA (**3**) is an intermediate in the biosynthesis of DHA (**2**) from EPA (**1**) [41,42,43], as shown in Scheme 5. EPA (**1**) is converted to n-3 DPA (**3**) by elongase enzymes. Further elongase and Δ^6^-desaturase activities on **3** yield the 24:6 n-3 fatty acid (**30**) via **31**, which is finally converted to DHA (**2**) by β-oxidation. The absence of the C4-C5 *Z*-configured double bond in n-3 DPA makes this PUFA incapable of biosynthesizing the RvD3, RvD4, and RvD6 n-3 DPA congeners since this double bond is involved in the biosynthesis of RvD3, RvD4, and RvD6. The naming of the n-3 DPA maresins, protectins, and resolvins is based on the chemically similar structures as the DHA-derived SPMs [34]. 

## 2. Stereoselective Syntheses of n-3 DPA-Derived SPMs

Since SPMs are biosynthesized only on the nano- to picogram scale, direct NMR analyses for their individual structural elucidations are not possible. Hence, mass spectrometry-based identification using multiple reacting monitoring (MRM) is therefore used to establish the structures from biological sources [8]. The inconvenience of this method is that it can only provide the basic structures without stereochemistry. Hence, matching the biogenic product with the product obtained by stereoselective total synthesis with defined stereochemistry is necessary to establish the complete stereochemical assignment of the SPMs. Specialized pro-resolving mediators are interesting biotemplates in drug development efforts with the aim to provide new anti-inflammatory agents without immunosuppressive effects [21,23]. The n-3 DPA (**3**)-derived SPMs are no exception [44]. 

The following sections provide an overview of the hitherto published syntheses of the various SPMs derived from n-3 DPA (**3**). In addition, an overview of the biological studies reported is also presented. 

### 2.1. Synthesis and Biological Studies of PD1_n-3 DPA_ (***11***)

**Scheme 6:**
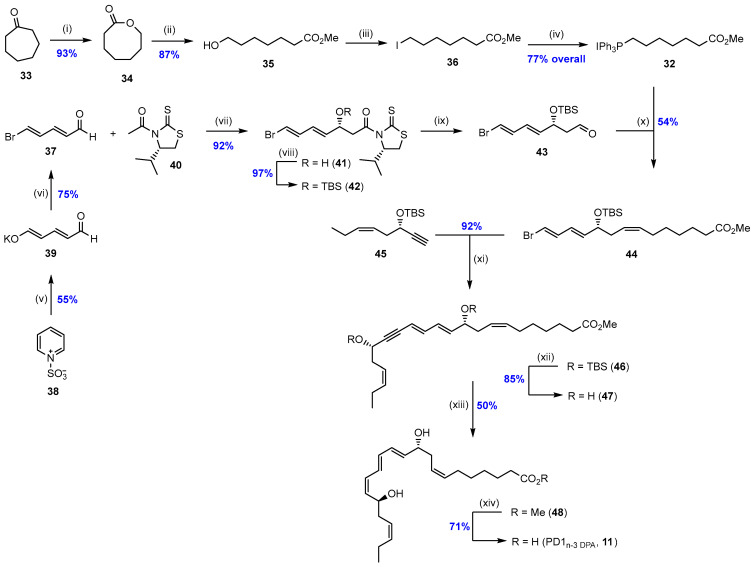
Stereoselective synthesis of PD1_n-3 DPA_ (**11**) [45]. Reagents and conditions: (i) *m*-CPBA, CH_2_Cl_2_; (ii) cat. H_2_SO_4_, MeOH; (iii) Ph_3_P, I_2_, imidazole, CH_2_Cl_2_; (iv) Ph_3_P, MeCN, Δ; (v) KOH (aq), −20 °C to rt; (vi) Br_2_, PPh_3_, CH_2_Cl_2_, 0 °C, then *p*-TsOH, Et_2_O; (vii) TiCl_4_, (*i*-Pr)_2_NEt, CH_2_Cl_2_, −78 °C; (viii) TBSOTf, 2,6-lutidine, CH_2_Cl_2_, −78 °C; (ix) DIBAL-H, CH_2_Cl_2_, −78 °C; (x) NaHMDs, THF, −78 °C; (xi) Pd(PPh_3_)_4_, CuI, Et_2_NH; (xii) TBAF, THF, 0 °C; (xiii) H_2_, Lindlar’s catalyst, EtOAc/pyridine/1-octene; (xiv) LiOH (aq), MeOH, 0 °C.

The first total synthesis of PD1_n-3 DPA_ (**11**) was reported in 2014 [45]. This was a convergent synthesis with Wittig salt **32**, aldehyde **43,** and alkyne **45** as key fragments (Scheme 6). The Wittig salt **32** was prepared in four steps from cycloheptanone (**33**). A Baeyer–Villiger oxidation of **33** yielded the lactone **34** in 93% yield, which was treated with catalytic amounts of H_2_SO_4_ in MeOH to yield the ring-opened hydroxy methyl ester **35** in 87% yield. An Appel reaction using Ph_3_P, I_2_, and imidazole in CH_2_Cl_2_ was next applied to convert the alcohol moiety in **35** to the corresponding iodide **36**. Finally, refluxing **36** with Ph_3_P in MeCN yielded the desired Wittig salt **32** in 77% yield over the two steps. For the synthesis of the key fragment **43**, the known aldehyde **37** was prepared as previously reported from commercially available pyridinium-1-sulphonate (**38**) using a two-step protocol [46,47]. The first treatment of salt **38** with aqueous potassium hydroxide at −20 °C yielded the glutaconaldehyde potassium salt **39,** which was transformed further with the Br_2_/PPh_3_ complex to aldehyde **37** in 41% yield over the two steps. Then, an Evans–Nagao acetate aldol with chiral auxiliary **40** and aldehyde **37** was executed, which yielded the aldol product **41** in 15.3:1 dr, according to the procedure of Olivio and coworkers [48]. Protection as TBS-ether **42** and reductive removal of the auxiliary yielded aldehyde **43**, as earlier reported in the literature [49,50]. Aldehyde **43** was then reacted in a highly *Z*-selective Wittig reaction with the ylide of **32** to furnish the desired *Z*-alkene **44** in 54% yield. The yield in this reaction was hampered by the elimination of the TBS-protected alcohol in **44** to yield an all-conjugated system. Alkyne **45** was prepared from commercially available 1-butyne and THP-protected (*S*)-glycidol, as reported earlier [51,52]. A Sonogashira cross-coupling reaction between the vinylic bromide in **44** and alkyne **45** was achieved to yield **46** an excellent 92% yield using catalytic amounts of Pd(PPh_3_)_4_ and CuI in Et_2_NH. Deprotection of the two silyl ethers in **46** was achieved using TBAF in THF at 0 °C, which afforded **47**. A *Z*-selective reduction of the internal alkyne in **47** was then carried out using Lindlar’s catalyst in a solvent system containing EtOAc/pyridine/1-octene (10:1:1) to obtain PD1_n-3 DPA_ methyl ester (**48**) in 50% yield. Finally, saponification of the methyl ester **48** using LiOH in H_2_O/MeOH (1:1) at 0 °C afforded the natural product **11** in 71% yield and chemical purity >98% based on HPLC chromatography. The longest linear sequence of this synthesis was 10 steps, with an overall yield of 9%. Matching experiments between synthetic and endogenous PD1_n-3 DPA_ (**11**) provided evidence for the absolute configuration to be (7*Z*,10*R*,11*E*,13*E*,15*Z*,17*S*,19*Z*)-10,17-dihydroxydocosa-7,11,13,15,19-pentaenoic acid (**11**). 

PD1_n-3 DPA_ (**11**) is the n-3 DPA SPM member that has been the subject of most biological studies. This SPM displays potent anti-inflammatory and pro-resolving bioactivities comparable to those of PD1 (**8**) in that it decreases neutrophil recruitment during peritonitis and increases macrophage phagocytosis of both zymosan A and apoptotic neutrophils [45]. A recent study revealed that PD1_n-3 DPA_ (**11**) regulated neuroinflammation and reduced weight loss and cognitive deficit during epileptogenesis, in addition to halting the ensuing epileptic seizures [53]. Another interesting study uncovered the protective effects of **11** against colitis and intestinal inflammation in mice [54]. 

### 2.2. Synthesis of PD2_n-3 DPA_ (***12***)

**Scheme 7:**
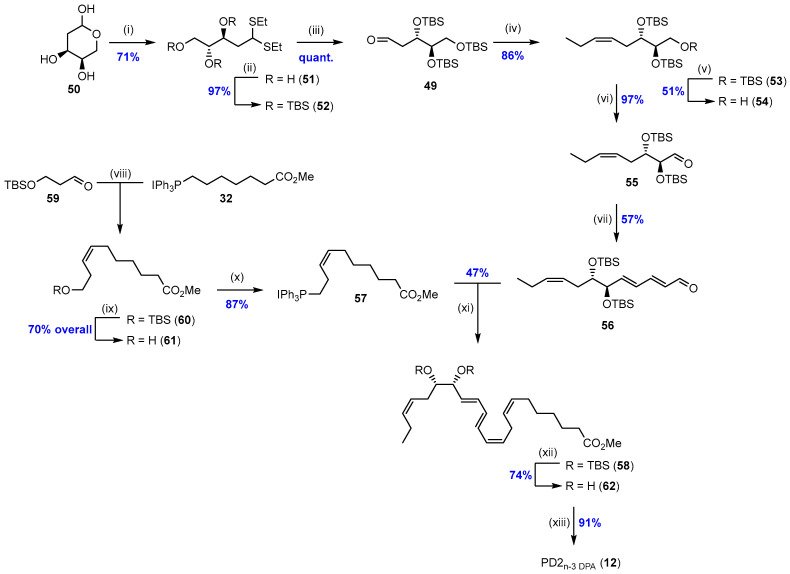
Stereoselective synthesis of PD2_n-3 DPA_ (**12**) [55]. Reagents and conditions: (i) EtSH, HCl, rt., 3 h; (ii) TBSOTf, 2,6-lutidine, 0 °C, 16 h; (iii) NBS, 2,6-lutidine, acetone/H_2_O, 0 °C, 1 h; (iv) NaHMDS, BrPh_3_P(CH_2_)_2_CH_3_, CH_2_Cl_2_, −78 °C to rt; (v) PTSA, EtOH/MeOH (1:1), −20 °C to rt; (vi) DMP, NaHCO_3_, CH_2_Cl_2_, rt; (vii) (triphenylphosphoranylidene)acetaldehyde, toluene, 95 °C; (viii) NaHMDS, HPMA, THF, then **59**, −78 °C; (ix) CSA, MeOH/CH_2_Cl_2_ (2:1); (x) PPh_3_, I_2_, imidazole, CH_2_Cl_2_, then Ph_3_P, MeCN, Δ; (xi) NaHMDS, THF, HMPA, −78 °C; (xii) TBAF, THF, 0 °C to rt; (xiii) LiOH, THF/MeOH/H_2_O (2:2:1), 0 °C.

The hitherto only reported total synthesis of PD2_n-3 DPA_ (**12**) was published in 2020 by Primdahl, Tungen, and Hansen [55]. This convergent synthesis relied on the two key fragments, aldehyde **56** and Wittig salt **57** (Scheme 7). For the synthesis of key aldehyde **56**, compound **49** [56] was prepared from 2-deoxy-D-ribose (**50**). Conversion of **50** into its thioacetal **51** was followed by global protection into **52** and cleavage of the thioacetal to afford aldehyde **49**. A *Z*-selective Wittig reaction of aldehyde **49** with the ylide of commercially available propyltriphenylphosphonium bromide, the latter obtained in situ after treatment with NaHMDS, afforded **53** (Scheme 7). Selective deprotection by PTSA in EtOH/MeOH at −20 °C revealed the primary alcohol **54**, which was further partially oxidized to **55** using Dess–Martin periodinane. Aldehyde **55** was then reacted in a double *E*-selective Wittig reaction with (triphenylphosphoranylidene)acetaldehyde in toluene at elevated temperature to yield the key fragment **56**. The other key fragment, Wittig salt **57**, was obtained by following a four-step sequence. Firstly, a *Z*-selective Wittig reaction between aldehyde **59** and the ylide of **32**, the latter obtained after reaction with NaHMDS, yielded *Z*-alkene **60**. Then, deprotection yielded alcohol **61,** and an Appel reaction and quaternization with triphenylphosphine afforded **57** (Scheme 7). The two key fragments, aldehyde **56** and Wittig salt **57,** were joined by a *Z*-selective Wittig reaction to afford compound **58**. The final steps included deprotection of the TBS-ethers in compound **58** with TBAF to yield the diol **62,** which was hydrolyzed to PD2_n-3 DPA_ (**12**). This convergent synthesis provided the target molecule PD2_n-3 DPA_ (**12**) in 10 steps (longest linear sequence) and 5% overall yield. As of today, no biological studies with **12** have been reported.

### 2.3. Synthesis and Biological Evaluations of MaR1_n-3 DPA_ (***15***)

**Scheme 8:**
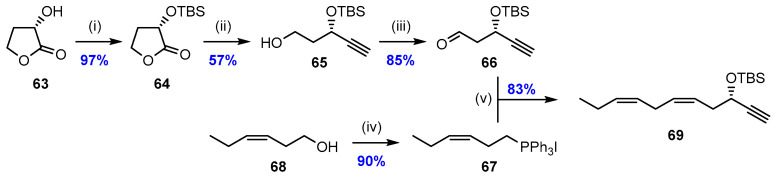
Synthesis of the ω-fragment **69** needed for the preparation of MaR1_n-3 DPA_ (**15**) [57]. Reagents and conditions: (i) TBSOTf, 2,6-lutidine, CH_2_Cl_2_, −78 °C; (ii) DIBAL-H, CH_2_Cl_2_, −78 °C, then LDA, TMSCHN_2_, THF, −78 °C; (iii) (COCl)_2_, DMSO, Et_3_N, CH_2_Cl_2_, −78 °C; (iv) I_2_, PPh_3_, imidazole, CH_2_Cl_2_, then PPh_3_, MeCN, Δ; (v) NaHMDS, HMPA, THF, −78 °C.

**Scheme 9:**
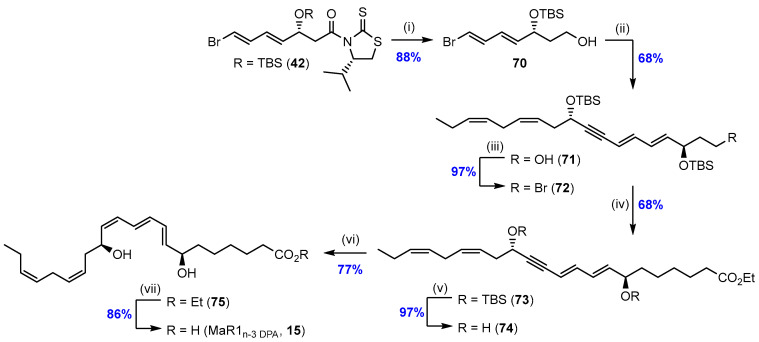
Final steps in the stereoselective synthesis of MaR1_n-3 DPA_ (**15**) [57]. Reagents and conditions: (i) LiBH_4_, Et_2_O, MeOH; (ii) Pd(PPh_3_)_4_, CuI, alkyne **69**, Et_2_NH; (iii) CBr_4_, PPh_3_, 2,6-lutidine, CH_2_Cl_2_; (iv) Pd-PEPPSI^TM^-IPr, LiCl, 4-ethoxy-4-oxobutylzinc bromide, THF, NMP; (v) TBAF, THF, 0 °C; (vi) Lindlar’s catalyst, H_2_, EtOAc, pyridine, 1-octene; (vii) LiOH, THF, MeOH, 0 °C.

The only synthesis of MaR1_n-3 DPA_ (**15**) was reported in 2014 [57]. This convergent synthesis relied on a Sonogashira reaction between the two key fragments alkyne **69** (Scheme 8) and vinyl bromide **70**, and a sp^3^–sp^3^ Negishi cross-coupling reaction between bromide **72** and 4-ethoxy-4-oxobutylzinc bromide (Scheme 9). The synthesis of alkyne **69** commenced with the protection of the alcohol moiety in commercially available (*S*)-(−)-α-hydroxy-γ-butyrolactone (**63**) using TBSOTf and 2,6-lutidine in CH_2_Cl_2_ to yield compound **64** in near quantitative yield, as shown in Scheme 8. Lactone **64** was then reduced to the corresponding lactol using DIBAL-H in CH_2_Cl_2_ at −78 °C, followed by a solvent switch to THF and reacted in a Colvin homologation reaction using LDA and trimethylsilyldiazomethane (TMSCHN_2_) to afford alcohol **65** in 57% isolated yield. Swern oxidation yielded aldehyde **66**, which was subjected to a *Z*-selective Wittig reaction with the ylide of **67**. This yielded the desired alkyne **69** in 83% yield. The Wittig salt **67** was prepared in 90% yield from commercially available (*Z*)-3-hexen-1-ol (**68**) over two steps using a literature procedure [58].

The vinyl bromide **70** was prepared by reduction of known **42** (see Scheme 6 for the synthesis of **42**) with LiBH_4_ in a solvent system containing Et_2_O and MeOH. A Sonogashira cross-coupling reaction of alkyne **69** and the vinylic bromide in **70** yielded the product **71** in an acceptable 68% yield. The primary alcohol in **71** was transformed to the corresponding bromide **72** to be further reacted in a sp^3^–sp^3^ Negishi cross-coupling reaction using the commercial palladium-based PEPPSI™-IPr catalyst, which yielded the alkyne **73**. This is an example of an early application of the sp^3^–sp^3^ Negishi cross-coupling reaction in the total synthesis of an advanced natural product. Removal of the two TBS-ethers in **73** using fluoride anions yielded the diol **74** in near quantitative yield. The internal alkyne in **74** was reduced in a highly *Z*-selective fashion by employing Lindlar’s catalyst in a mixed solvent system containing EtOAc, pyridine, and 1-octene under a hydrogen atmosphere. Finally, saponification of the ethyl ester yielded the desired MaR1_n-3 DPA_ (**15**) in 86% yield and chemical purity >98% based on HPLC analyses. This convergent synthesis yielded MaR1_n-3 DPA_ (**15**) over 11 steps (longest linear sequence) and 12% overall yield. 

Matching experiments between synthetic and human macrophage MaR1_n-3 DPA_ (**15**) demonstrated that the synthetic material co-elutes with the naturally occurring **15**, establishing the absolute configuration to be (7*S*,8*E*,10*E*,12*Z*,14*S*,16*Z*,19*Z*)-7,14-dihydroxydocosa-8,10,12,16,19-pentaenoic acid. MaR1_n-3 DPA_ (**15**) was next assayed for its potential to enhance human macrophage efferocytosis, a highly important pro-resolution hallmark. The results provided clear evidence for the potent bioactions of **15** and its corresponding ethyl ester in stimulating efferocytosis of apoptotic human neutrophils by macrophages [57].

### 2.4. Synthesis of MaR2_n-3 DPA_ (***16***)

**Scheme 10:**
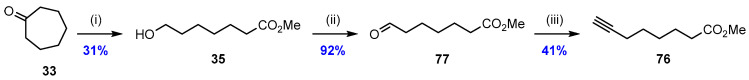
Synthesis of terminal alkyne **76** needed for the synthesis of MaR2_n-3 DPA_ (**16**) [59]. Reagents and conditions: (i) *m*-CPBA, CH_2_Cl_2_, then MeOH, H_2_SO_4_; (ii) DMP, NaHCO_3_, CH_2_Cl_2_; (iii) dimethyl(1-diazo-2-oxopropyl) phosphonate, K_2_CO_3_, MeOH.

**Scheme 11:**
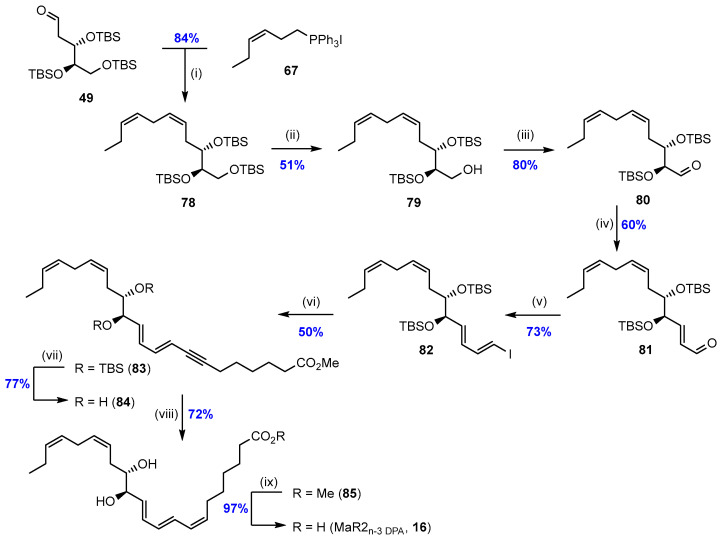
Final steps in the stereoselective synthesis of MaR2_n-3 DPA_ (**16**) [59]. Reagents and conditions: (i) NaHMDS, CH_2_Cl_2_, −78 °C; (ii) *para*-toluene sulfonic acid, MeOH, −20 °C; (iii) DMP, NaHCO_3_, CH_2_Cl_2_; (iv) (triphenylphosphoranylidene)acetaldehyde, toluene, Δ; (v) CrCl_2_, dioxane, THF, CHI_3_, 0 °C; (vi) CuI, Pd(PPh_3_)_4_ (5 mol%), alkyne **76**, Et_2_NH; (vii) TBAF, THF; (viii) Lindlar’s catalyst (Pd/CaCO_3_), EtOAc/pyridine/1-octene, H_2_ (g); (ix) LiOH, H_2_O, MeOH, 0 °C.

A stereoselective total synthesis of MaR2_n-3 DPA_ (**16**) was reported in 2020 [59]. The key fragments in this synthesis were alkyne **76** (Scheme 10), aldehyde **49,** and Wittig salt **67** (Scheme 11). Alkyne **76** was synthesized in a four-step sequence, starting with a Bayer–Villiger oxidation of commercially available cycloheptanone (**33**), which was followed by Fischer esterification of the corresponding lactone of **33**, as shown in Scheme 10, to yield compound **35** in 31% yield. Next, partial oxidation of the primary alcohol moiety in **35** using the Dess–Martin periodinane reagent afforded aldehyde **77** in 92% yield. A Seyferth–Gilbert homologation reaction, using the Ohira–Bestmann reagent, with aldehyde **77** produced the terminal alkyne **76** in 41% yield. 

Aldehyde **49** was prepared as previously reported [56] (see Scheme 7). A *Z*-selective Wittig reaction with the ylide of Wittig salt **67**, the latter obtained in situ after reacting **67** with NaHMDS at −78 °C and aldehyde **49**, yielded *Z*-alkene **78**. Selective deprotection of the primary TBS-ether in **78** using *para*-toluene sulfonic acid (PTSA) in MeOH at −20 °C revealed the primary alcohol **79**, which was further oxidized to the corresponding aldehyde **80** using the Dess–Martin periodinane (DMP) reagent. Next, an *E*-selective Wittig between aldehyde **80** and (triphenyl-phosphoranylidene)acetaldehyde in toluene at elevated temperature afforded the α,β-unsaturated aldehyde **81** in 60% yield. A Takai olefination reaction was then performed on aldehyde **81** to yield the *E*,*E*-vinylic iodide **82** in 73% isolated yield after chromatographic purifications. A palladium-mediated Sonogashira cross-coupling reaction between vinylic iodide **82** and alkyne **76** yielded the key intermediate **83** in 50% yield. Diol **84** was prepared by deprotection of the two TBS-ethers in **83** using TBAF in THF. A Lindlar hydrogenation protocol afforded the desired methyl ester **85** in 72% yield and chemical purity >95% based on HPLC analysis. Finally, a mild hydrolysis of the methyl ester **85** yielded the desired natural product MaR2_n-3 DPA_ (**16**) in 97% isolated yield. Matching experiments of synthetic and endogenous **16** revealed that the right stereoisomer was prepared, thus establishing the absolute configuration of MaR2_n-3 DPA_ (**16**) to be (7*Z*,9*E*,11*E*,13*R*,14*S*,16*Z*,19*Z*)-13,14-dihydroxydocosa-7,9,11,16,19-pentaenoic acid (**16**). MaR2_n-3 DPA_ (**16**) has not been utilized in any biological studies. 

### 2.5. Synthesis and Biological Evaluations of RvD1_n-3 DPA_ (***19***)

**Scheme 12:**
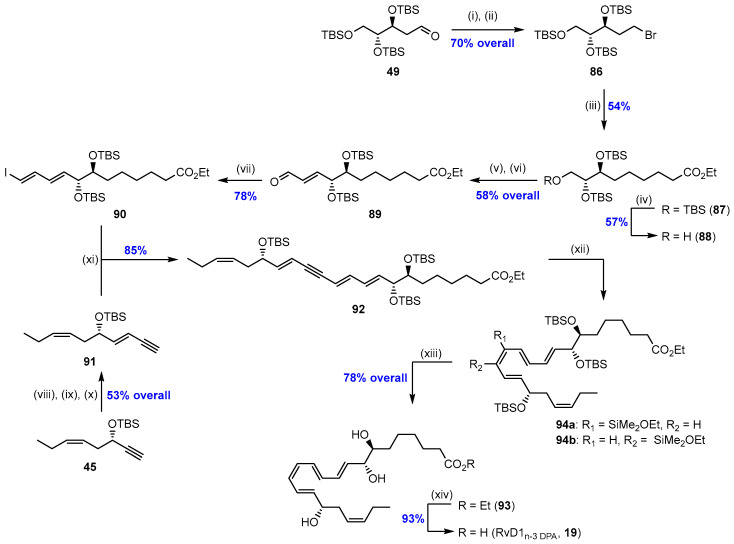
Stereoselective total synthesis of RvD1_n-3 DPA_ (**19**) [56]. Reagents and conditions: (i) NaBH_4_, MeOH, 0 °C, 1 h; (ii) CBr_4_, PPh_3_, 2,6-lutidine, −10 °C, 4 h; (iii) BrZn(CH_2_)_3_CO_2_Et, Pd-PEPPSI^TM^-IPr, LiBr, THF/DMI, 40 °C, 2 h; (iv) PTSA, MeOH, −20 °C, 1 h; (v) DMP, NaHCO_3_, CH_2_Cl_2_, rt, 20 h; (vi) Ph_3_P=CHCHO, toluene, 95 °C, 18 h; (vii) CrCl_2_, CHI_3_, THF/dioxane, 0 °C to rt, 1.5 h; (viii) Cp_2_ZrCl_2_, DIBAL-H, then I_2_, THF, 0 °C, 30 min; (ix) TMSCCH, piperidine, CuI, Pd(PPh_3_)_2_Cl_2_, THF, rt, 3 h; (x) K_2_CO_3_, MeOH, rt., 2 h; (xi) Pd(PPh_3_)_4_, CuI, benzene, Et_2_NH, rt, 18 h; (xii) Karstedt’s cat., Me_2_SiHOEt, toluene, rt, 16 h; (xiii) TBAF, THF, 0 °C to rt., 20 h; (xiv) LiOH, THF, MeOH, H_2_O, 0 °C, 4 h.

In 2019, a total synthesis of RvD1_n-3 DPA_ (**19**) was reported [56]. This convergent synthesis relied on a Sonogashira cross-coupling reaction between the two key fragments, vinyl iodide **90** and alkyne **91** (Scheme 12). Vinyl iodide **90** was prepared in six steps from the known aldehyde **49** (Scheme 7). First, aldehyde **49** was reduced to the corresponding alcohol with NaBH_4_ in MeOH, followed by an Appel halogenation to yield the bromide **86**. This bromide was subjected to a sp^3^-sp^3^ Negishi cross-coupling reaction with 4-ethoxy-4-oxobutylzinc bromide using the palladium-based PEPPSI^TM^-IPr catalyst to furnish compound **87** in 54% isolated yield. Selective removal of the primary TBS-ether using PTSA revealed the primary alcohol **88**. Partial oxidation of **88** using Dess–Martin periodinane, followed by an *E*-selective Wittig reaction, yielded the α,β-unsaturated aldehyde **89** in 58% yield over the two steps. A Takai olefination reaction converted aldehyde **89** to the vinyl iodide **90**. The other key fragment, alkyne **91**, was prepared from the previously prepared compound **45** [51,52] in a three-step sequence, including a zirconation/iodination reaction, a Sonogashira coupling, and a deprotection reaction. A Sonogashira cross-coupling was then performed to unite **90** and **91**. The product herein, **92**, was next assumed to be reduced in a *Z*-selective manner; however, both the trusted Boland and Lindlar reductions on **92** and its triol failed to yield the desired ethyl ester **93**. The Karstedt alkyne hydrosilylation/protodesilylation protocol [60] was then utilized to convert internal alkyne **92** to the corresponding *Z*-alkene, which gratefully yielded, via **94a** and **94b**, the desired RvD1_n-3 DPA_ ethyl ester (**93**) in 78% yield over the two steps and with chemical purity > 97% based on HPLC analysis. A mild saponification of the ethyl ester yielded RvD1_n-3 DPA_ (**19**). Metabololipidomics LC-MS/MS experiments were used to determine if the synthetic and authentic material of RvD1_n-3 DPA_ (**19**) matched. Identical retention time and co-elution of synthetic and authentic **19** provided evidence for the right stereoisomer to be synthesized, thus establishing the absolute configuration of **19** to be (7*S*,8*R*,9*E*,11*E*,13*Z*,15*E*,17*S*,19*Z*)-7,8,17-trihydroxydocosa-9,11,13,15,19-pentaenoic acid. 

The synthetic material of RvD1_n-3 DPA_ (**19**) showed potent agonism toward the human receptor GPR32 in an impedance assay, and this SPM displayed nanomolar anti-inflammatory, pro-resolution, and anti-bacterial effects [56].

### 2.6. Synthesis and Biological Evaluations of RvD2_n-3 DPA_ (***20***)

**Scheme 13:**
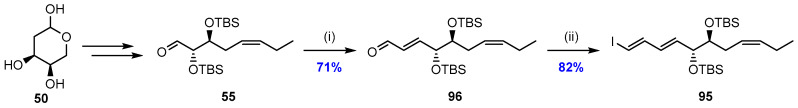
Synthesis of vinyl iodide **95** [61]. Reagents and conditions: (i) (triphenylphosphoranylidene)acetaldehyde, toluene, 95 °C; (ii) CrCl_2_, CHI_3_, THF/dioxane, 0 °C to rt. Details for the preparation of **55** is given in reference [55].

**Scheme 14:**
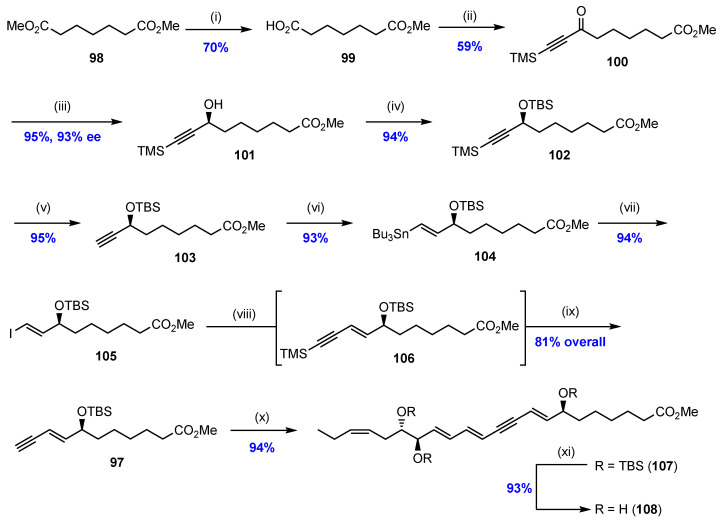
Synthesis of internal alkyne **97** and coupling of fragments [61]. Reagents and conditions: (i) NaOH (aq), THF, then HCl (aq), 0 °C; (ii) (COCl)_2_, DMF (cat), CH_2_Cl_2_, 0 °C to rt, then AlCl_3_, BTMSA, CH_2_Cl_2_, 0 °C to rt; (iii) (*S*)-Alpine-borane, THF to ~neat, −10 °C to rt; (iv) TBSCl, imidazole, CH_2_Cl_2_; (v) K_2_CO_3_, MeOH, 0 °C to rt; (vi) Bu_3_SnH, AIBN (16 mol%), benzene, 80 °C; (vii) I_2_, CH_2_Cl_2_; (viii) TMSCCH, CuI (12 mol%), Pd(PPh_3_)_2_Cl_2_ (5 mol%), Et_3_N, THF, 0 °C to rt; (ix) K_2_CO_3_, MeOH, 0 °C to rt; (x) vinyl iodide **95**, CuI (9 mol%), Pd(PPh_3_)_4_ (3 mol%), Et_2_NH, benzene; (xi) TBAF, THF, 0 °C to rt.

The stereoselective total synthesis of RvD2_n-3 DPA_ (**20**) was reported as a convergent synthesis, relying on a Sonogashira cross-coupling reaction between the two key fragments vinyl iodide **95** and alkyne **97** (Scheme 13 and Scheme 14) [61]. For the synthesis of vinyl iodide **95,** aldehyde **55** was first prepared from commercially available and cheap 2-deoxy-D-ribose (**50**) as previously reported in the literature [55] (Scheme 7), and then **55** was reacted in an *E*-selective Wittig reaction with commercially available (triphenylphosphoranylidene)acetaldehyde to yield α,β-unsaturated aldehyde **96** in 71% yield. A Takai olefination reaction on **96** furnished the desired vinyl iodide **95** in an excellent 82% yield. 

For the preparation of the terminal alkyne **97** needed for the subsequent Sonogashira cross-coupling reaction with **95**, commercially available and cheap diester **98** was first selectively hydrolyzed using aqueous NaOH in THF at a lowered temperature. The carboxylic acid **99** was then converted to its acid chloride in situ, followed by a Friedel–Crafts acylation with bis(trimethylsilyl)acetylene (BTMSA) to yield **100** in acceptable 59% yield. The Midland (*S*)-Alpine-borane reagent was then applied to synthesize (*S*)-alcohol **101** in 93% enantiomeric excess and 95% yield. The alcohol in **101** was protected using TBSCl and imidazole in CH_2_Cl_2_ to yield **102**, followed by removal of the TMS group using K_2_CO_3_ in MeOH to obtain terminal alkyne **103**. Hydrostannylation of **103** yielded **104**, which was subjected to an in situ iodination protocol to prepare vinyl iodide **105**. Vinyl iodide **105** was in fact first reported in 2020 by Rodriguez and Spur for the total synthesis of RvT1 (**26**) and RvT4 (**29**) [62]. The greatest difference between the two syntheses of **105** was the use of the Midland Alpine-borane reagent for the asymmetric reduction of the acetylenic ketone herein rather than the ruthenium-catalyzed asymmetric reduction. Also, the availability of reagents was crucial for choosing different reaction conditions for the synthesis of vinyl iodide **105** herein. Next, a Sonogashira cross-coupling between **105** and trimethylsilylacetylene yielded crude **106,** which was directly subjected to a TMS-deprotection step to obtain terminal alkyne **97** in 81% yield. Vinyl iodide **95** and alkyne **97** were reacted in a Sonogashira cross-coupling reaction with Pd(PPh_3_)_4_ (3 mol%) and CuI (9 mol%) as the catalysts of choice to afford the internal alkyne **107**. The protection groups were then removed with TBAF in THF to yield triol **108** in an excellent 93% yield. 

**Scheme 15:**
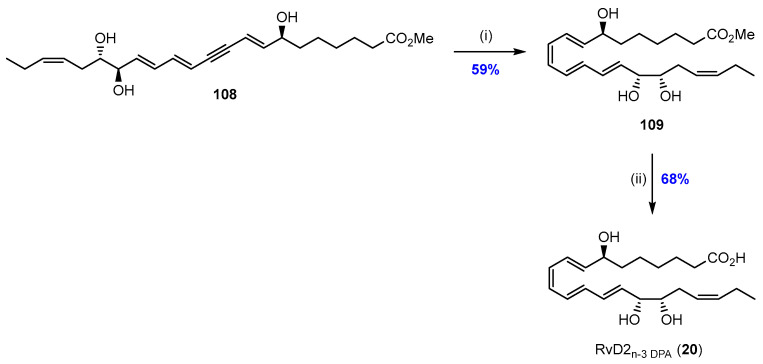
Final steps in the total synthesis of RvD2_n-3 DPA_ (**20**) using the Näf reduction protocol. Reagents and conditions: (i) Zn/KCN, 1-propanol/H_2_O, then TMSCHN_2_, toluene/MeOH; (ii) LiOH (aq), THF, −78 to 4 °C.

For the *Z*-selective reduction of the internal alkyne in **108**, several different strategies were attempted [63,64] that proved problematic. No product formation was observed using the Lindlar catalyst. The Karstedt platinum-catalyzed alkyne hydrosilylation/protodesilylation protocol [60] was successful for the synthesis of structurally similar RvD1_n-3 DPA_ (**19**) [56]; hence, this reaction was attempted next for the *Z*-selective reduction of **107**. Using this procedure, the protodesilylation step could also remove the silyl ethers in one pot. Unfortunately, the two-step reaction afforded a mixture of the desired product **109** and inseparable by-products. HPLC analysis revealed a chemical purity of disappointingly 81% after extensive purification by flash chromatography utilizing different combinations of eluent systems. Finally, a *Z*-selective hydrogenation protocol using potassium cyanide and zinc in 1-propanol/H_2_O [65] yielded the natural product **20** (Scheme 15). However, due to issues in the purification step, re-esterification with TMS-diazomethane in toluene/MeOH was needed, which afforded the RvD2_n-3 DPA_ methyl ester (**109**) in 59% yield over the two steps and in >96% chemical purity based on HPLC analysis. Hydrolysis of **109** to RvD2_n-3 DPA_ (**20**) was performed just prior to matching experiments and biological testing due to the inherent chemical-sensitive nature of this SPM. MRM LC-MS/MS matching experiments were conducted that revealed that the synthetically produced material **20** was indeed identical to that of biologically produced RvD2_n-3 DPA_ (**20**). Also, these studies confirmed the structure of **20** to be (7*S*,8*E*,10*Z*,12*E*,14*E*,16*R*,17*S*,19*Z*)-7,16,17-trihydroxydocosa-8,10,12,14,19-pentaenoic acid (**20**). 

RvD2_n-3 DPA_ (**20**) potently increased the uptake of the Gram-positive *S. aureus* bacteria in macrophages [61]. The effects were dose-dependent, between 0.01 and 10 nM. Moreover, using the same doses, macrophages pretreated with synthetic **20** also showed a statistically significant increase in the digestion of zymosan A bioparticles, a type of macromolecules derived from the yeast wall of *Saccharomyces cerevisiae,* thus providing evidence for its anti-fungal activity as well [61]. The clearance of such pathogens and inflammatory molecules by macrophages is a key step in the resolution phase of inflammation [66].

### 2.7. Synthesis and Biological Evaluations of RvD5_n-3 DPA_ (***21***)

**Scheme 16:**
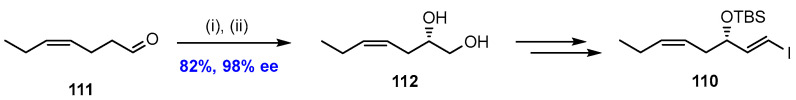
Synthesis of vinyl iodide **110**. (i) D-proline, PhNO, CHCl_3_, 0 °C to rt; (ii) NaBH_4_, EtOH, then Zn, AcOH, 82%, 98% ee. Details for the preparation of **110** is given in reference [68].

**Scheme 17:**
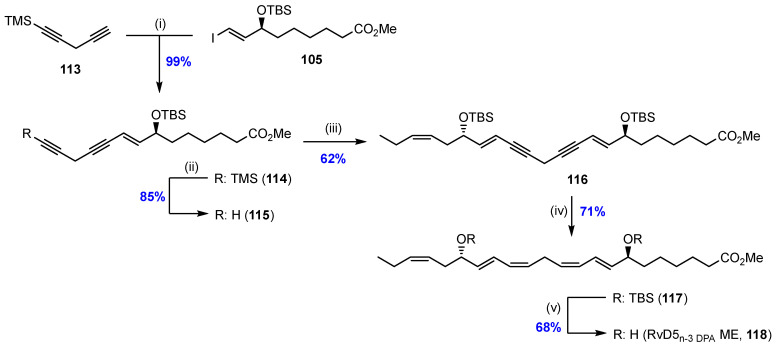
Synthesis of terminal alkyne **115** and the final steps for making RvD5_n-3 DPA_ methyl ester (**118**) [67]. Reagents and conditions: (i) Pd(PPh_3_)_2_Cl_2_ (5 mol%), CuI (12 mol%), Et_3_N, THF; (ii) AgNO_3_, KCN, THF, EtOH, H_2_O; (iii) Pd(PPh_3_)_2_Cl_2_ (5 mol%), CuI (11 mol%), Et_3_N, vinyl iodide **110**, THF; (iv) Pd/BaSO_4_, quinoline, EtOAc; (v) cat. AcCl, MeOH.

The stereoselective synthesis of RvD5_n-3 DPA_ (**21**) was reported very recently [67]. This convergent synthesis relied on the two key fragments, vinyl iodide **110** (Scheme 16) and alkyne **115** (Scheme 17), which were combined in a Sonogashira cross-coupling reaction. The synthesis of vinyl iodide **110** was achieved in seven steps starting from commercially available and affordable (*Z*)-4-heptenal (**111**), as previously reported [68]. The most prominent step herein was the Macmillan organocatalytic α-oxyamination reaction, which afforded the diol **112** in 82% yield and 98% ee. Diastereomerically pure **110** was obtained from diol **112** after an *E*-selective Takai olefination reaction as the most pivotal step.

For the synthesis of the other fragment, alkyne **115**, the known vinyl iodide **105** [61] was reacted in a Sonogashira cross-coupling reaction with commercially available **113** to afford the coupled product **114** in quantitative yields (Scheme 17). Next, compound **114** was reacted with AgNO_3_ and KCN to gently remove the TMS-protection group and reveal the terminal alkyne **115**. Alkyne **115** was further reacted in another palladium-mediated Sonogashira coupling with vinyl iodide **110** to yield the carbon backbone **116** in an acceptable 62% yield. The alkyne **115** is most likely highly unstable and prone to rapid decomposition, hence the slightly modest yield in this step, although using 2.5 equivalents of **115**. For the *Z*-selective reduction of the two internal alkynes in **116**, the Lindlar hydrogenation protocol was first attempted, but no reduction of the triple bonds was observed. A *Z*-selective hydrogenation protocol utilizing Pd/BaSO_4_/quinoline was then applied, which showed rapid and selective conversion of the two internal alkynes to the respective *Z*-alkenes and furnished compound **117** in 71% yield. Finally, a mild removal of the TBS-ethers using catalytic amounts of acetyl chloride in dry MeOH afforded the RvD5_n-3 DPA_ methyl ester (**118**) in 68% yield and chemical purity of 97% based on HPLC analysis. This convergent synthesis achieved the methyl ester of RvD5_n-3 DPA_ in 8% overall yield over 12 steps (longest linear sequence).

Hydrolysis of the methyl ester **118** to RvD5_n-3 DPA_ (**21**) was conducted just prior to analyses due to the inherent chemically sensitive nature of this SPM. Firstly, matching experiments with synthetic and biogenic **21** provided evidence for the right stereoisomer to be synthesized. Next, agonism studies of RvD5_n-3 DPA_ (**21**) with the human receptor GPR101 were measured. These results confirmed the nanomolar agonism of RvD5_n-3 DPA_ (**21**) toward this GPCR. Additionally, the anti-inflammatory, pro-resolution, and anti-bacterial effects of **21** [67] were evaluated and confirmed [69].

### 2.8. Synthesis and Biological Actions of RvT1 (***26***) and RvT4 (***29***)

**Scheme 18:**
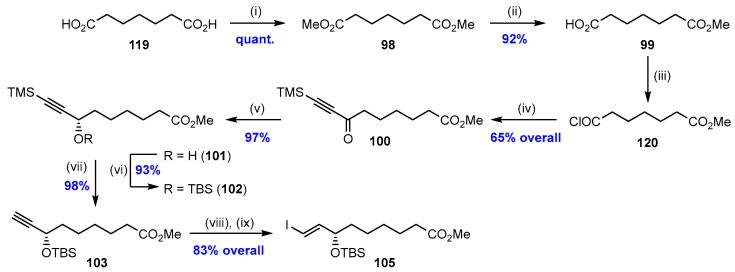
Preparation of compound **105** needed for the total synthesis of RvT4 (**29**) and RvT1 (**26**) by Rodriguez and Spur [62]. Reagents and conditions: (i) TMSCl, 2,2-dimethoxypropane, CH_3_OH, rt; (ii) porcine pancreatic lipase, 0.05 M phosphate buffer pH 7, 1 N NaOH; (iii) (COCl)_2_, cat. DMF, CH_2_Cl_2_, rt; (iv) BTMSA, AlCl_3_, CH_2_Cl_2_, 0 °C; (v) RuCl[(*S*,*S*)-TsDPEN](*p*-cymene), cetrimonium bromide (CTAB), HCOONa, H_2_O, EtOAc, rt; (vi) TBSCl, imidazole, 4-DMAP, CH_2_Cl_2_, 0 °C to rt; (vii) K_2_CO_3_, Na_2_SO_4_, CH_3_OH, rt; (viii) (Bu)_3_SnH, AIBN, benzene, reflux; (ix) I_2_, CH_2_Cl_2_, 0 °C.

Rodriguez and Spur reported the only total synthesis of RvT1 (**26**) and RvT4 (**29**) so far in 2020 [62]. These two SPMs are structurally quite similar (see Scheme 4), and the authors came up with a clever solution where RvT1 (**26**) could be produced from synthetic RvT4 (**29**) by a lipoxygenation of the latter. One of the two key fragments in this synthesis, vinylic iodide **105,** was prepared in a nine-step sequence (Scheme 18), starting from dicarboxylic acid **119**. The diacid **119** was first esterified to yield the diester **98**, followed by selective hydrolysis of one of the methyl esters using porcine pancreatic lipase. The resulting product **99** was next transformed into the corresponding acid chloride **120**, which was reacted in a Friedel–Crafts acylation with bis(trimethylsilyl)acetylene (BTMSA) in the presence of AlCl_3_ to yield the propargyl ketone **100** in 65% yield over the two steps. A Noyori asymmetric hydrogenation protocol was applied to introduce the (*S*)-alcohol at C_7_ in an excellent 97% yield and >94% ee. TBS-protection of the alcohol, followed by TMS-deprotection of the terminal acetylene, produced compound **103** in 91% yield over the two steps. Finally, a standard hydrostannylation/iodination protocol afforded the vinyl iodide **105** in 83% yield.

**Scheme 19:**
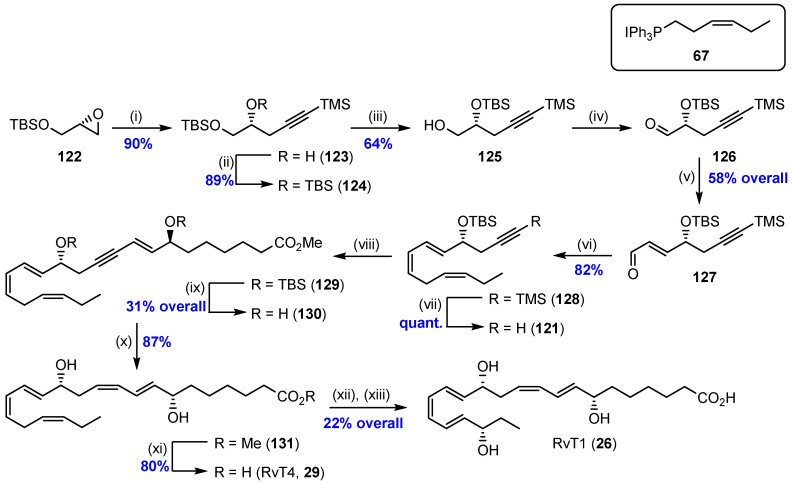
Preparation of the ω-fragment **121** and coupling of fragments to complete the synthesis of RvT4 (**29**). RvT1 (**26**) was prepared from enzymatic hydroxylation of RvT4 (**29**) [62]. Reagents and conditions: (i) Trimethylsilylacetylene, *n*-BuLi, BF_3_·Et_2_O, THF, –78 °C; (ii) TBSCl, imidazole, 4-DMAP, CH_2_Cl_2_, 0 °C to rt; (iii) CSA, CH_2_Cl_2_/MeOH 1/1, 0 °C; (iv) Dess–Martin periodinane, CH_2_Cl_2_, rt; (v) (Triphenylphosphoranylidene)acetaldehyde, MeCN, 30 °C; (vi) **67**, *n*-BuLi, THF, –78 °C to 0 °C; (vii) K_2_CO_3_, CH_3_OH, rt; (viii) **105**, Pd(PPh_3_)_4_, CuI, piperidine, benzene, rt; (ix) CH_3_COCl, MeOH, 0 °C to rt; (x) Zn(Cu/Ag), CH_3_OH, H_2_O, 40–45 °C; (xi) 1 N LiOH, MeOH/H_2_O 1/1, 0 °C to rt; (xii) lipoxidase type I-B from soybean, 0.01 M borate buffer pH 10.7, rt; (xiii) TCEP-HCl, rt.

The other key fragment in this synthesis, terminal alkyne **121**, was prepared starting from commercially available and optically pure glycidol derivative **122,** which was reacted with trimethylsilylacetylene, *n*-BuLi, and BF_3_·Et_2_O to yield the secondary alcohol **123**, as shown in Scheme 19. The secondary alcohol in **123** was then protected using TBSCl, imidazole, and 4-DMAP, followed by selective deprotection of the primary TBS-ether using CSA in CH_2_Cl_2_/MeOH. Treatment of the resulting alcohol **125** with Dess–Martin periodinane yielded the corresponding aldehyde **126,** which was reacted in an *E*-selective Wittig reaction with (triphenylphosphoranylidene)acetaldehyde to obtain the α,β-unsaturated aldehyde **127**. A *Z*-selective Wittig reaction between **127** and known phosphorane **67** provided compound **128** in 82% yield. Removal of the TMS-protection group revealed the terminal acetylene **121,** which was next reacted with vinyl iodide **105** in a Sonogashira cross-coupling reaction, providing the whole carbon skeleton of the target compounds. The two TBS-protection groups in **129** were then removed using acetyl chloride in MeOH to yield diol **130**. A Boland reduction protocol was applied to selectively reduce the internal triple bond in **130** to predominantly yield the *Z*-olefin **131**. Hydrolysis of the methyl ester **131** afforded the desired natural product RvT4 (**29**). To obtain RvT1 (**26**), RvT4 (**29**) was subjected to a lipoxygenation using lipoxidase type I-B from soybean. After the reduction of the resulting hydroperoxide with tris(2-carboxyethyl)phosphine hydrochloride (TCEP-HCl), pure RvT1 (**26**) was obtained after HPLC purification and desalting. 

Patients with rheumatoid arthritis have an increased risk of developing cardiovascular diseases, including atherosclerosis. To this end, RvT4 (**29**) recently proved to enhance macrophage cholesterol efflux in arthritic mice to reduce vascular diseases and thus limit morbidity in inflammatory arthritis [70]. 

### 2.9. Synthesis of RvT2 (***27***) and Biological Actions of the RvTs

**Scheme 20:**
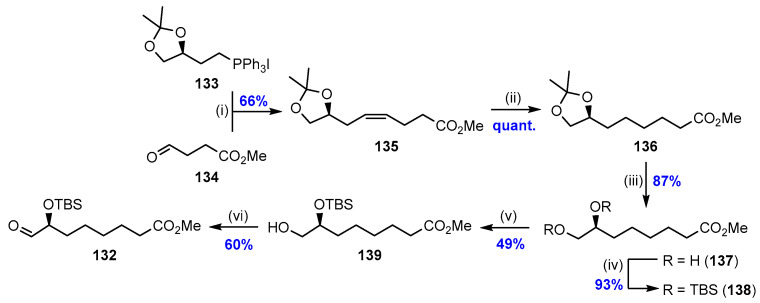
Synthesis of compound **132** needed for the preparation of RvT2 (**27**) by Rodriguez and Spur [71]. Reagents and conditions: (i) *n*-BuLi, THF, −78 °C to −20 °C; (ii) H_2_, Pt/C, hexane/methyl acetate 9/1, rt; (iii) 1 N HCl, MeOH, 0 °C; (iv) TBSCl, imidazole, 4-DMAP, CH_2_Cl_2_, 0 °C to rt; (v) CSA, CH_2_Cl_2_/MeOH 1/1, 0 °C; (vi) Dess–Martin periodinane, CH_2_Cl_2_, rt.

Rodriguez and Spur have disclosed the hitherto only synthesis of RvT2 (**27**) [71]. The synthesis commenced with the preparation of aldehyde **132** (Scheme 20). Firstly, commercially available *S*-(−)-1,2,4-butanetriol was transformed to the corresponding crystalline phosphonium iodide **133** and thus reacted with aldehyde **134** in a Wittig reaction to afford olefin **135**. The double bond was next reduced using platinum on carbon under a hydrogen atmosphere to yield the saturated compound **136** in quantitative yield. Removal of the acetonide-protection group using diluted HCl, followed by TBS-protection, yielded the desired silylated compound **138**. Selective removal of the TBS-ether of the primary alcohol to yield **139** was achieved in an acceptable 49% yield. Finally, oxidation of the primary alcohol **139** using Dess–Martin periodinane afforded compound **132**. 

**Scheme 21:**
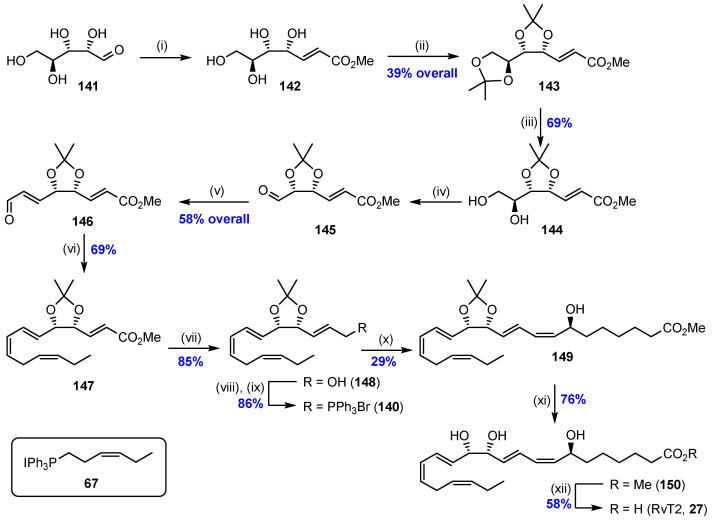
Synthesis of compound **140** and coupling of fragments to furnish RvT2 (**27**) [71]. Reagents and conditions: (i) methyl (triphenylphosphoranylidene)acetate, benzoic acid, 1,4-dioxane, reflux; (ii) acetone/2,2-dimethoxypropane (2/1), *p*-toluenesulfonic acid, rt; (iii) amberlyst^®^ 15, MeOH/H_2_O 9/1, rt; (iv) NaIO_4_, CH_3_OH/H_2_O 10/4, 0 °C to rt; (v) (triphenylphosphoranylidene)acetaldehyde, benzene, rt; (vi) **67**, *n*-BuLi, THF, −78 °C to −20 °C; (vii) DIBAL-H, CH_2_Cl_2_, −78 °C to −20 °C; (viii) CBr_4_, Ph_3_P, CH_2_Cl_2_, 0 °C; (ix) Ph_3_P, CH_2_Cl_2_, 0 °C to rt; (x) **132**, KHMDS, THF, −78 °C to −20 °C; (xi) 2 N HCl, MeOH, 0 °C to rt, 76%; (xii) 1 N LiOH, MeOH/H_2_O 2/1, 0 °C to rt.

The synthesis of the other key fragment in this synthesis, the Wittig salt **140**, began with the reaction of L-(+)-ribose (**141**) with methyl (triphenylphosphoranylidene)acetate, as shown in Scheme 21. This *E*-selective Wittig reaction yielded **142**, which was used directly in the next step to yield the diacetonide-protected compound **143**. Selective removal of the terminal acetonide using amberlyst^®^ 15 yielded compound **144**. Cleavage of the diol using NaIO_4_ afforded aldehyde **145,** which was reacted in an *E*-selective Wittig reaction with (triphenylphosphoranylidene)acetaldehyde to yield **146** and then further subjected to a *Z*-selective Wittig reaction with the ylide prepared from phosphonium iodide **67**. The product herein, **147**, was reduced with DIBAL-H at low temperature to convert the ester moiety to the corresponding alcohol. The resulting alcohol **148** was then transformed to the Wittig salt **140** and reacted with aldehyde **132** in a new *Z*-selective Wittig reaction to provide **149** in 29%, containing the carbon backbone of the target compound. Finally, acidic removal of the acetonide-protection group, followed by basic hydrolysis of the methyl ester, provided the natural product RvT2 (**27**). Also, the synthesis of the 13(*R*)-epimer of RvT2 (**27**) was reported by Rodriguez and Spur by following the same synthetic route as for **27**, starting from d-(−)-arabinose instead of l-(+)-ribose (**141**) [71]. 

Recently, the SPMs **26**–**29** were reported to reduce neutrophil extracellular traps (NETs) in human blood [72]. The formation of NETs is primarily through a cell death process called NETosis [73] and is a way for neutrophils to protect the host against invading pathogens. NETs can trap microbes [74]; however, excessive formation is known to be a source of collateral tissue damage in the pathology of an array of diseases [75,76,77,78,79,80]. This phenomenon is especially known in SARS-CoV-2 infections [81] and acute respiratory distress syndrome (ARDS) [82]. Hence, the role of the RvTs in decreasing NETosis could be utilized to find a new approach for treating such infections in the future. As of today, no details on the total synthesis of RvT3 (**28**) have been reported. 

### 2.10. Synthesis and Biological Actions of the ω-22 Monohydroxylated Metabolite 22-OH-PD1 (***151***)

**Scheme 22:**
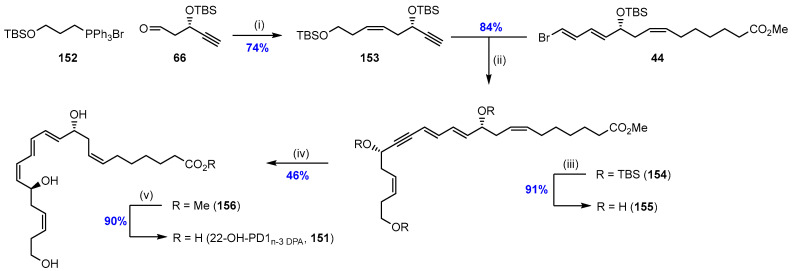
Synthesis of the ω-22 monohydroxylated metabolite **151** [83]. Reagents and conditions: (i) NaHMDS, THF, HMPA, −78 °C; (ii) Pd(PPh_3_)_4_, CuI, Et_2_NH, rt; (iii) TBAF, THF, −78 °C; (iv) Zn(Cu/Ag), MeOH/H_2_O, rt; (v) LiOH, MeOH, H_2_O, 0 °C. The synthesis of compound **44** is presented in Scheme 6.

At the current time, few reports exist on the further metabolism of n-3 DPA-derived SPMs. However, the further metabolism of **11** has been studied, which showed that the monohydroxy metabolite named 22-OH-PD1_n-3 DPA_ (**151**) was formed in human serum and neutrophils (Scheme 22). The Hansen group exploited the similarity with PD1_n-3 DPA_ (**11**) to synthesize its ω-oxidation further metabolite **151** [83]. Known aldehyde **66** [57] was reacted in a *Z*-selective Wittig reaction with the ylide of commercially available **152**, the latter obtained after reaction with NaHMDS, to obtain *Z*-olefin **153** in 74% yield. Compound **153** was next reacted in a palladium-mediated Sonogashira cross-coupling reaction with compound **44** to nicely yield the coupled product **154**. The three TBS-ethers in **154** were then removed using excess TBAF to afford triol **155,** which was further subjected to a Boland reduction protocol to yield 22-OH-PD1_n-3 DPA_ methyl ester (**156**) in an acceptable 46% yield. Finally, a saponification of the methyl ester in **156** yielded the metabolite **151** in 90% yield and 94% chemical purity (based on HPLC). 

Since SPMs are formed in nano- to picogram amounts at the site of injury, SPM metabolites are even more challenging to isolate and characterize. Hence, LC/MS-MS data were attained that showed that the biosynthetic and synthetic materials of **151** matched data from MRM experiments [83]. Biosynthetic studies with human neutrophils and human monocytes revealed in both experiments the direct formation of 22-OH-PD1_n-3 DPA_ (**151**) from PD1_n-3 DPA_ (**11**) [83]. Moreover, studies adding n-3 DPA to human neutrophils also allowed the detection of the metabolite **151** and its precursor PD1_n-3 DPA_ (**11**) [83].

## 3. Conclusions

n-3 DPA is a PUFA that has gained an increased interest in biomedical and life science research [84]. Biosynthetic studies in the presence of this PUFA and several oxygenase enzymes enabled the discovery of seven resolvins, three maresins, and two protectins [85], whereas ten of these have been prepared and confirmed by stereoselective total synthesis [45,55,56,57,59,61,62,67,71]. However, based on the chemical structure of n-3 DPA and the catalytic mechanisms of LOXs, several additional new DPA-derived SPMs are envisioned. Among those, sulfido-conjugated n-3 DPA-derived SPMs, similar to their original congeners, should be formed since epoxides are intermediates [36,37]. Moreover, the n-3 DPA-derived families of SPMs should also be attractive as substrates for receptor and metabolism studies, where less knowledge is available at this point. As of today, only the metabolite of PD1_n-3 DPA_ (**11**), named 22-OH-PD1_n-3 DPA_ (**151**), has been reported and studied [83]. The biological evaluations of simpler chemical synthetic analogs of the 12 n-3 DPA-derived SPMs known will also be of future interest. Some studies have emerged recently [86,87]. However, success in such endeavors is dependent on stereoselective synthesis of the native SPMs highlighted herein, but also the synthesis of isomers [88] and analogs [86], in particular since SPMs are produced in nanogram amounts in living systems, making NMR studies for their exact structural elucidation impossible to perform [89].

## Data Availability

Not applicable.
